# SG-APSIC1157: The attributable mortality and excess length of stay associated with third-generation cephalosporin-resistant Enterobacterales bloodstream infections—A prospective cohort study in Suva, Fiji

**DOI:** 10.1017/ash.2023.73

**Published:** 2023-03-16

**Authors:** Michael Loftus, Tracey Young-Sharma, Allen Cheng, Adam Jenney, Sue Lee, Ravi Naidu, Anton Peleg, Andrew Stewardson

**Affiliations:** Monash University, Melbourne, Australia; Colonial War Memorial Hospital, Suva, Fiji; Monash University, Melbourne, Australia; Monash University, Melbourne, Australia; Monash University, Melbourne, Australia; Colonial War Memorial Hospital, Suva, Fiji; Monash University, Melbourne, Australia; Monash University, Melbourne, Australia

## Abstract

**Objectives:** Although antimicrobial resistance (AMR) disproportionately affects low- and middle-income countries (LMICs), primary clinical data on AMR burden from LMICs are lacking, particularly from the Pacific Islands. We adapted recent World Health Organization methodology to measure the impact of third-generation cephalosporin (3GC) resistance on mortality and excess length of hospital stay among inpatients with Enterobacterales bloodstream infection (BSI) in Fiji. **Methods:** We conducted a prospective cohort study of inpatients with Enterobacterales BSIs at Colonial War Memorial Hospital in Suva. We collected demographic, clinical, and microbiological data, and we stored bacterial isolates for confirmatory testing and molecular genomics in Melbourne, Australia. We employed cause-specific Cox proportional hazards models to estimate the effect of 3GC-resistance on hazard of in-hospital mortality and discharge alive (competing outcomes), and we used multistate modelling to estimate the excess length of hospital stay associated with 3GCR. **Results:** From July 2020 to February 2021, we identified 162 consecutive Enterobacterales BSIs, and 66 (40.7%) were 3GC resistant. The crude mortality rates for patients with 3GC-susecptible and 3GC-resistant BSIs were 16.7% (16 of 96) and 30.3% (20 of 66), respectively. Also, 3GC resistance was not associated with either in-hospital mortality (aHR, 1.67; 95% CI, 0.80–3.49) or discharge alive (aHR, 0.75; 95% CI, 0.50–1.12). However, patient comorbidities and acuity of illness were associated with in-hospital mortality. Furthermore, 3GC-resistance was associated with an increased length of stay of 2.6 days (95% CI, 2.5–2.8). Overall, 3GC-resistance was more common among patients with hospital-associated than community-acquired infection, but genomics did not identify clonal transmission. **Conclusions:** Among patients with Enterobacterales BSIs, mortality was relatively high, and 3GC resistance was common. Also, 3GC resistance was associated with increased hospital length of stay but not with in-hospital mortality after adjusting for potential confounders. Accurate estimates of the burden of AMR are important, especially from LMICs. Such knowledge can inform policy decisions, guide allocation of limited resources, and assist the evaluation of future interventions to address AMR.

